# Supplementary material for: LncRNA EPR controls epithelial proliferation by coordinating Cdkn1a transcription and mRNA decay response to TGF-β
Source: Nat Commun. 2019 Apr 29;10:1969. doi: 10.1038/s41467-019-09754-1 (PMC6488594; doi:10.1038/s41467-019-09754-1)
Supplement: Supplementary file 1 — Supplementary Information [file 41467_2019_9754_MOESM1_ESM.pdf]

Rossi *et al*. "Long noncoding RNA EPR controls epithelial cell proliferation by coordinating *Cdkn1a* transcription and mRNA decay in response to TGF-β".

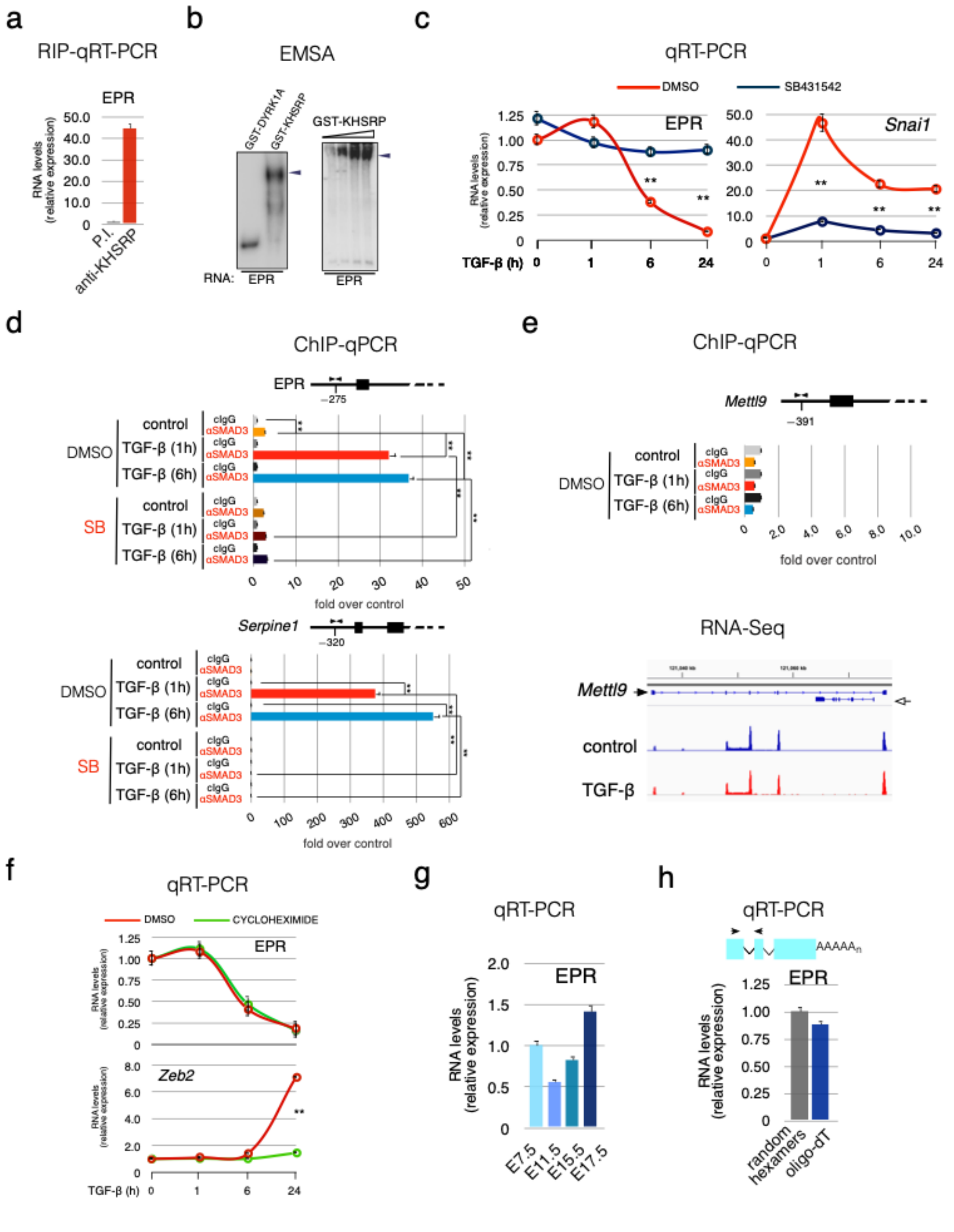

Rossi et al. Supplementary Figure 1

Supplementary Figure 1. EPR interacts with KHSRP and its expression is modulated by TGF-β signaling. (Related to Figure 1).

(a) Total extracts were prepared from NMuMG cells and immunoprecipitated as indicated. RNA was purified from immunocomplexes and analyzed by qRT-PCR to quantify EPR levels. P.I. stands for Pre-Immune serum. (b) Electrophoretic mobility shift assays (EMSA) performed using either recombinant DYRK1A (200 nM, negative control) or KHSRP (200 nM) (both fused with GST) and ^{32}P-labeled RNA corresponding to nucleotides 246-407 of EPR (left panel). Interaction between recombinant KHSRP (50–300 nM) and ^{32}P-labeled EPR RNA (right panel). Representative autoradiograms are displayed. Arrowhead points to the KHSRP-EPR complex. (c) NMuMG were serum-starved (2% FBS, 16h), pre-treated for 1 hour with 1 µM SB431542 and, then, treated with 1 ng/ml TGF-β for the indicated times or left untreated (time 0). The expression of EPR and *Snai1* (positive control for the SB431542 compound efficiency) was analyzed by qRT-PCR. (d) Chromatin prepared from NMuMG cells serum-starved, pre-treated with either DMSO (solvent) or 1 µM SB431542 (SB) and then either treated with 1 ng/ml TGF-β for the indicated times or left untreated (control) was immunoprecipitated using either normal rabbit IgG (cIgG) or affinity-purified anti-SMAD3 rabbit polyclonal antibody. The association of SMAD3 with either the EPR promoter (upper panel) or *Serpine1* promoter (lower panel) was quantified by qPCR using specific primers (indicated as arrowheads in the schematics above each panel). (e) SMAD3 does not interact with the negative control promoter of *Mettl9* (upper panel) whose expression is not affected by TGF-β as revealed by RNA-Seq experiments (lower panel). Solid arrow points to the *Mettl9* gene while open arrow points to an unrelated gene —present in the same locus— which is not expressed in NMuMG cells. (f) NMuMG were serum-starved (2% FBS, 16h), pre-treated for 1 hour with either DMSO (solvent) or 5 µM Cycloheximide and, then, treated with 1 ng/ml TGF-β for the indicated times or left untreated (time 0). EPR and *Zeb2* (also known as SIP1, positive control for cycloheximide treatment) expression was analyzed by qRT-PCR. (g) qRT-PCR analysis of EPR expression in total RNA extracted from mouse embryos at the indicated gestational ages. (h) qPCR analysis of EPR expression evaluated using a set of primers annealing with exons 1 and 2 of EPR precursor RNA (in order to amplify spliced EPR). Total RNA from NMuMG cells was retro-transcribed using either random hexamers or oligo-dT (as indicated, see also the Methods Section). The position of primers that have been used is indicated in the schematic on the top.

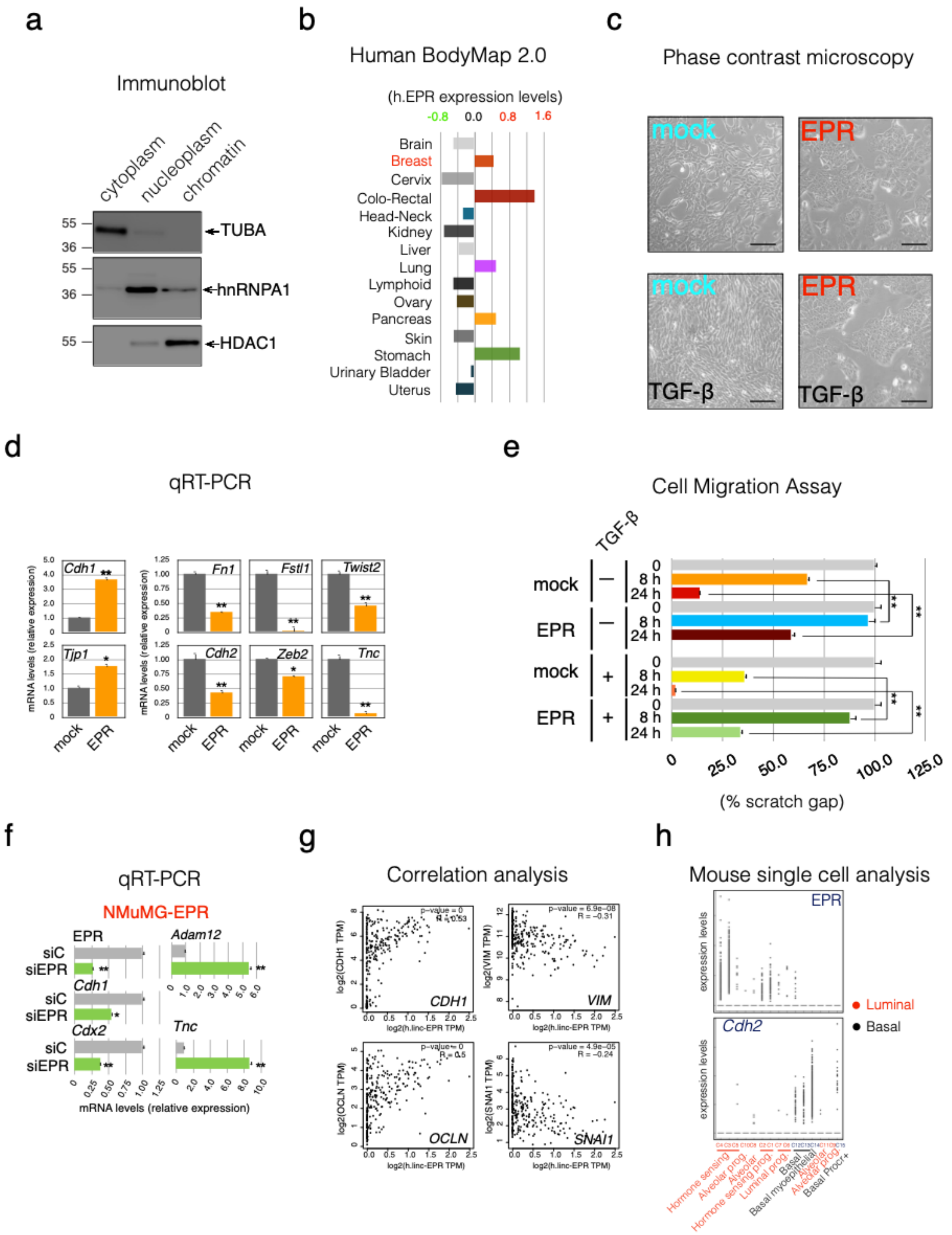

Rossi et al. Supplementary Figure 2

<u>Supplementary Figure 2.</u> (Related to Figure 1).

(a) Immunoblot analysis of cell fractions obtained as outlined in the Methods Section and used for experiments depicted in Fig. 1c. The indicated antibodies were used. The position of molecular mass markers is indicated on the left. Representative gels are shown. TUBA is also known as Tubulin Alpha. (b) The expression levels of h.EPR in multiple human tissue were

extracted from the Illumina Human Body Map 2.0 data. Data were median centered to distinguish the tissues with high expression of h.EPR. Breast is in red. (c) Phase contrast microscopy of either mock or EPR-overexpressing (EPR) NMuMG cells that were serum-starved and then either treated with TGF-β or left untreated for 24 hr. Scale bars: 100 μm. (d) qRT-PCR analysis of the indicated transcripts in either mock or EPR-overexpressing (EPR) NMuMG cells. (e) Scratch wound healing assays. Scratch wounds were introduced into confluent monolayers of either mock or EPR-overexpressing NMuMG cells. Cultures were either treated with TGF-β (for either 8 hours or 24 hours) or left untreated (0). Cultures were photographed and the width of the wound was measured at 0 hr, 8 hr, and 24 hr after the scratch was made. The percentage of the scratch gap area for each culture condition was plotted. (f) qRT-PCR analysis of the indicated transcripts in NMuMG cells overexpressing EPR and transiently transfected with either control siRNA (siC) or siRNAs to EPR (siEPR). (g) Pearson correlation analysis between the expression levels of h.EPR and the indicated human transcript as calculated by the GEPIA website (http://gepia.cancer-pku.cn) using as input breast normal samples from the TCGA dataset and normal mammary gland tissues. Expression levels are plotted as Transcripts per million (TPM, log-scale). The value of Pearson correlation coefficient (R) and the p-value are presented. (h) Bioinformatics analysis of datasets derived from single cell RNA-Seq performed in mice (https://marionilab.cruk.cam.ac.uk/mammaryGland/). Cells with Luminal phenotype are indicated in red while cells with Basal phenotype are in black.

The values of qRT-PCR and cell migration assays experiments shown are averages (±SEM) of three independent experiments performed in triplicate. Statistical significance: $*p < 0.01$, $**p < 0.001$ (Student's t-test).

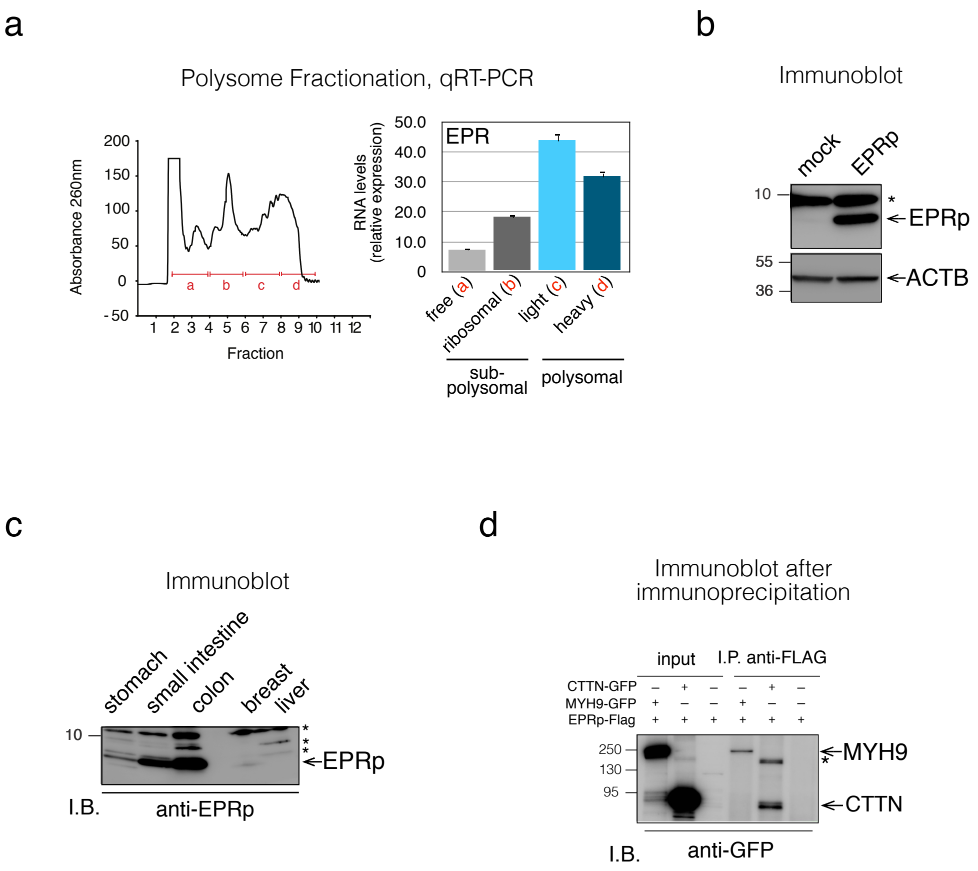

Rossi et al. Supplementary Figure 3

Supplementary Figure 3. Endogenous EPRp is expressed in epithelial tissues and interacts with cytoskeletal and junctional proteins. (Related to Figure 2).

(a) Left panel. Representative example of polysome profiles obtained from cytoplasmic lysates of NMuMG cells fractionated by sucrose gradients. The relative absorbance of the various fractions is indicated. Four pools of fractions corresponding to free RNA + 40S (a), 60S + 80S

(b), light polysomes (c), heavy polysomes (d) were prepared and processed for RNA extraction and qRT-PCR. Right panel. Relative expression of EPR across the four pools of sucrose gradient, cytoplasmic lysate fractions. The average relative expression and the standard error of three replicates are plotted. (b) Immunoblot analysis of total cell extracts from either mock or EPR-transfected HEK-293 cells. Asterisks mark nonspecific immunoreactivity. ACTB is also known as Actin Beta. (c) Immunoblot analysis of total extracts from the indicated mouse tissues. Asterisks mark nonspecific immunoreactivity. (d) Co-immunoprecipitation of FLAG-tagged EPRp and distinct cytoskeletal proteins (as indicated) in total extracts from HEK-293 cells transiently co-transfected with EPRp-FLAG and the indicated GFP-fused proteins. Asterisks mark nonspecific immunoreactivity.

For Immunoblots, the indicated antibodies were used; the position of molecular mass markers is indicated on the left and representative gels are shown.

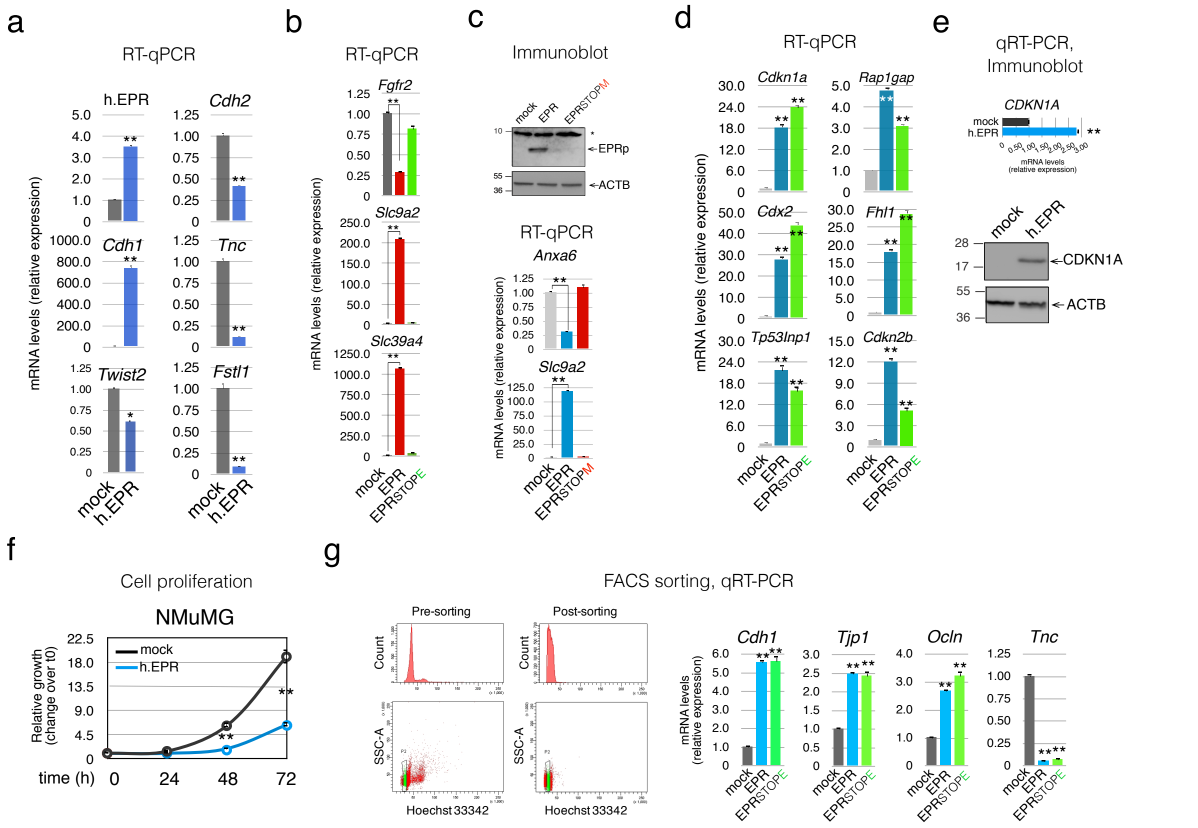

Rossi et al. Supplementary Figure 4

<u>Supplementary Figure 4. Gene expression changes induced by human EPR and EPRp synthesis in NMuMG cells</u>. (Related to Figure 3).

(a) qRT-PCR analysis of the indicated transcripts in either mock or human EPR (h.EPR) -overexpressing NMuMG cells. (b) qRT-PCR analysis of the indicated transcripts in either mock, EPR or EPRSTOPE-overexpressing NMuMG cells. (c) Immunoblot analysis of HEK-293 cells transiently transfected with either the empty vector, EPR or EPRSTOPM (top panel). qRT-PCR analysis of the indicated transcripts in either mock, EPR or EPRSTOPM-overexpressing NMuMG cells (bottom panel). (d) qRT-PCR analysis of the indicated transcripts in either mock, EPR or EPRSTOPE-overexpressing NMuMG cells. (e) qRT-PCR analysis of the *Cdkn1a* mRNA levels in either mock or human EPR (h.EPR) -overexpressing NMuMG cells (top panel). Immunoblot analysis of either mock or human EPR (h.EPR) -overexpressing NMuMG cells (bottom panel). (f) Cell proliferation analysis (assayed by Crystal violet staining) of either mock or human EPR (h.EPR) -overexpressing NMuMG cells. (g) Sorting of mock, EPR- or EPRSTOPE- overexpressing NMuMG cells in the G1 phase of the cell cycle. Cells were stained by Hoechst 33342 and processed using a BD Aria II cytometer. The pre-sorting cell cycle profile (top left), gating (green area in the dot plot, bottom left), and purity checked by

post-sorting sample re-run (center panels) are shown for one replicate of the mock sample; SSC-A is for side scatter area. qRT-PCR analysis of the sorted cells (right panels).

The values of qRT-PCR experiments shown are averages (±SEM) of three independent experiments performed in triplicate. The values of cell proliferation experiments (panel e) are averages (±SEM) of three independent experiments performed in triplicate. Statistical significance: $^{**}p < 0.001$ (Student's t test). Please note that the statistical significance presented in panels (d) and (g) results from comparison of gene expression levels in either EPR or EPRSTOPE-overexpressing cells compared with mock cells. For Immunoblots, the indicated antibodies were used; the position of molecular mass markers is indicated on the left and representative gels are shown. ACTB is also known as Actin Beta.

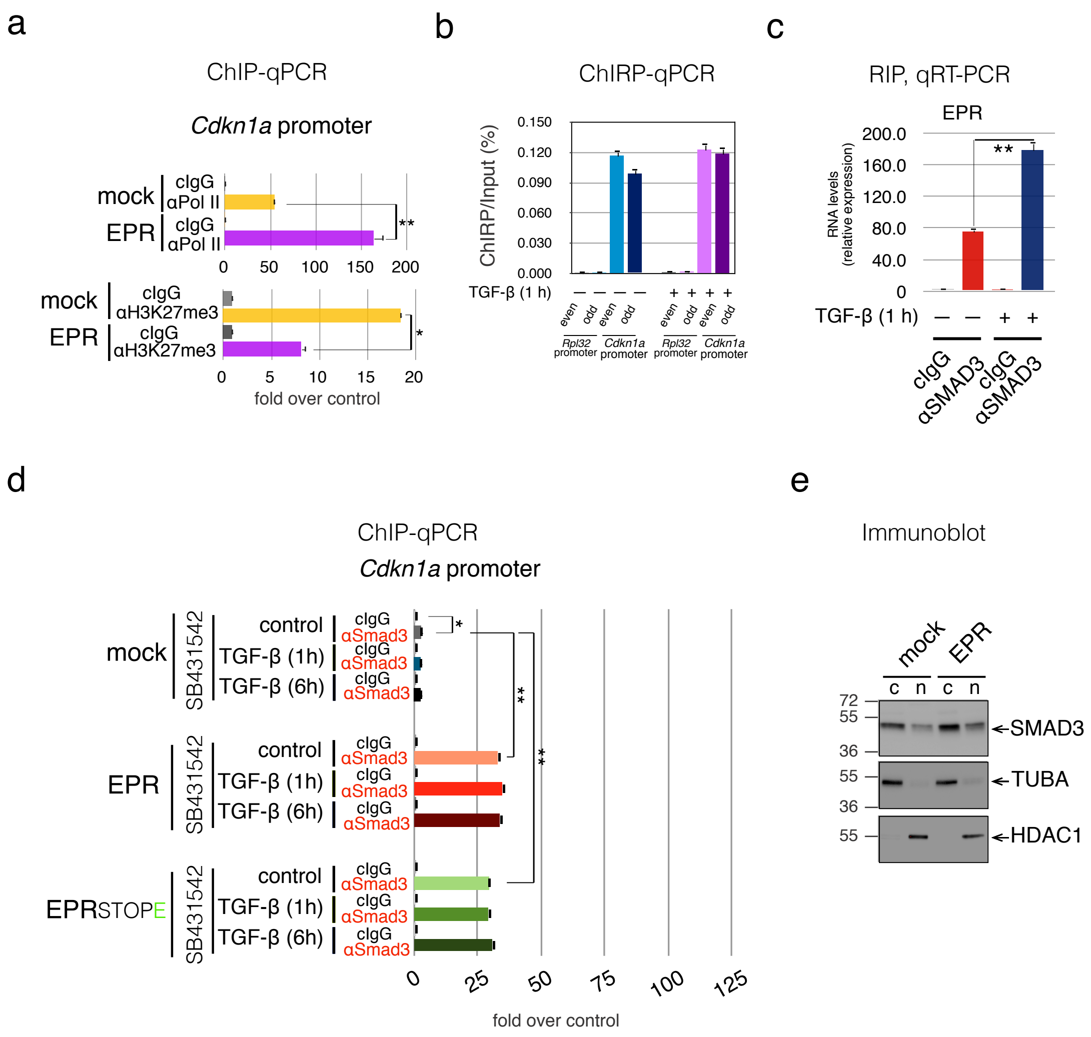

Rossi et al. Supplementary Figure 5

Supplementary Figure 5. EPR affects transcription of *Cdkn1a* gene in NMuMG cells. (Related to Figures 4 and 5).

(a) Top panel, chromatin prepared from either mock or EPR-overexpressing NMuMG cells was immunoprecipitated using either normal mouse IgG (cIgG) or mouse monoclonal anti-PolII

antibody. The association of PolII with *Cdkn1a* promoter was verified by qPCR using specific primers. Bottom panel, chromatin prepared from either mock or EPR-overexpressing NMuMG cells was immunoprecipitated using either normal rabbit IgG (cIgG) or affinity purified rabbit anti-H3K27me3 antibody. The association of H3K27me3 with *Cdkn1a* promoter was verified by qPCR using specific primers. (b) NMuMG cells were serum-starved (2% FBS, 16h) and either treated with TGF-β (10 ng/ml) for 1 hour (+) or left untreated (-). ChIRP analyses was performed using cell lysates and either even or odd EPR probe sets. Both input and purified DNA were analyzed by qPCR using primers designed to amplify either *Rpl32* (negative control) or *Cdkn1a* promoters. Values are averages (±SEM) of three independent experiments performed in triplicate. (c) NMuMG cells were serum-starved (2% FBS, 16h) and either treated with TGF-β (10 ng/ml) for 1 hour (+) or left untreated (-). Total extracts were prepared and immunoprecipitated as indicated. RNA was purified from immunocomplexes and analyzed by qRT-PCR to quantify EPR levels. (d) Chromatin was prepared from either mock, EPR- or EPRSTOPE- overexpressing NMuMG cells serum-starved (2% FBS, 16h), pre-treated with SB431542 for 1 hour and either treated with TGF-β (1 ng/ml) for the indicated times or left untreated (control). Chromatin was immunoprecipitated using either normal rabbit IgG (cIgG) or affinity-purified anti-SMAD3 rabbit polyclonal antibody. The association of SMAD3 with *Cdkn1a* promoter was verified by qPCR using specific primers. (e) Immunoblot analysis of either cytoplasmic (c) or nuclear (n) cell extracts from either mock or EPR-overexpressing NMuMG cells. The indicated antibodies were used; the position of molecular mass markers is indicated on the left and representative gels are shown. TUBA is also known as Tubulin Alpha.

The values of qRT-PCR and qPCR experiments shown are averages (±SEM) of three independent experiments performed in triplicate. Statistical significance: *$p < 0.01$, **$p < 0.001$ (Student's t-test).

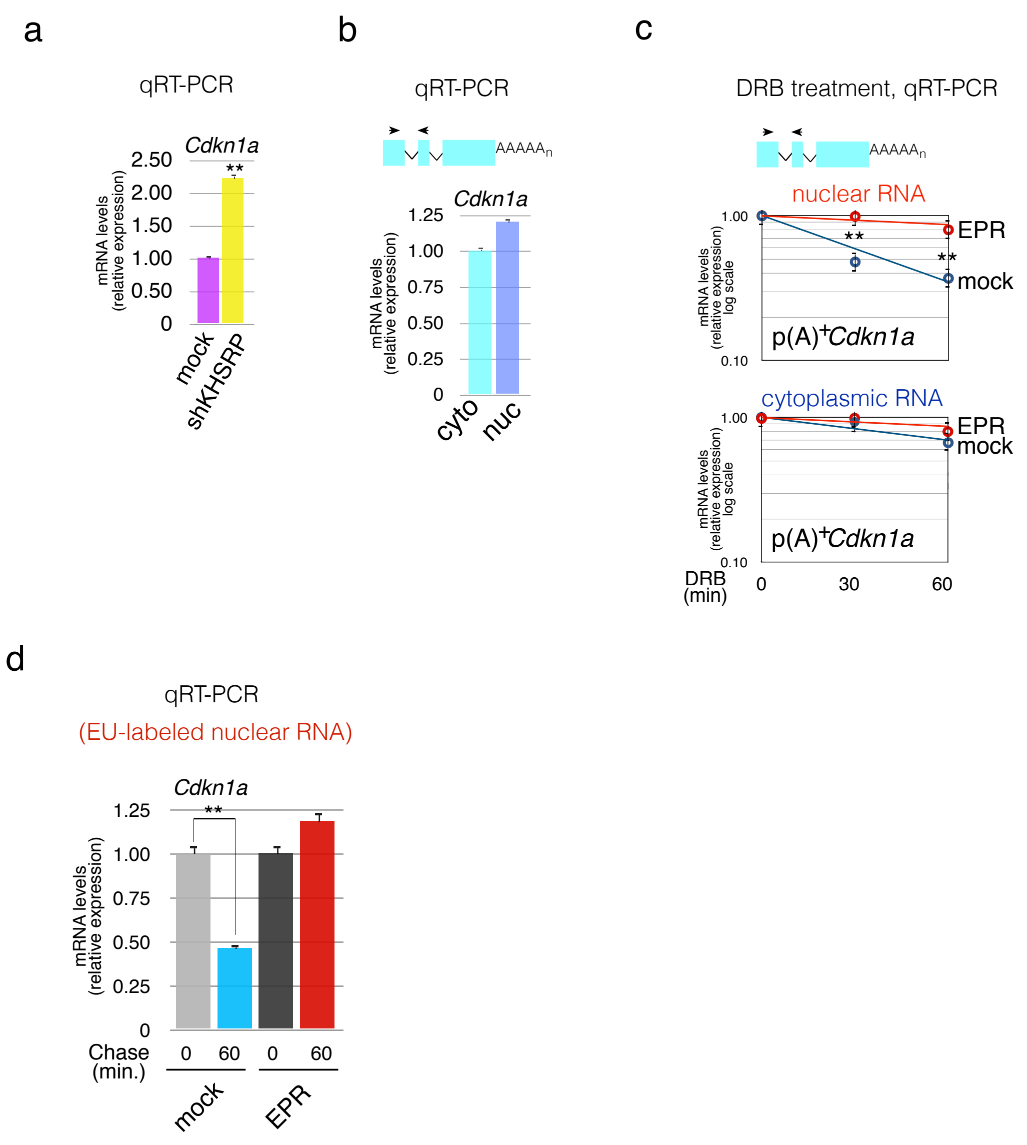

Rossi et al. Supplementary Figure 6

<u>Supplementary Figure 6. EPR affects mRNA decay of *Cdkn1a* gene in NMuMG cells</u>. (Related to Figures 4 and 5).

(a) qRT-PCR analysis of total RNA isolated from either mock or shKHSRP NMuMG cells. Please note that NMuMG mock cells used for these experiments differ from those presented throughout this report and have been described in Puppo *et al.*[15]. (b) qRT-PCR analysis of

polyadenylated and spliced *Cdkn1a* mRNA isolated from either cytoplasmic (cyto) or nuclear (nuc) extracts of NMuMG cells. The position of primers that have been used is indicated in the schematic on the top. Total RNA from NMuMG cells was retro-transcribed using oligo-dT. (c) Either mock or EPR-overexpressing NMuMG cells were treated with 100 μM 5,6-Dichlorobenzimidazole 1-β-D-ribofuranoside (DRB). Nuclear (top panel) and cytoplasmic (bottom panel) RNAs were isolated at different times (as indicated) and analyzed by qRT-PCR to quantify polyadenylated and spliced *Cdkn1a* mRNA levels. The position of primers that have been used is indicated in the schematic on the top. (d) Run-off analysis of EU-labeled nuclear RNA prepared from either mock-transfected (mock) or EPR-overexpressing (EPR) NMuMG cells (chase time 0 and 60 minutes).

The values of qRT-PCR and qPCR experiments shown are averages (±SEM) of three independent experiments performed in triplicate. Statistical significance: **$p < 0.001$ (Student's t test).

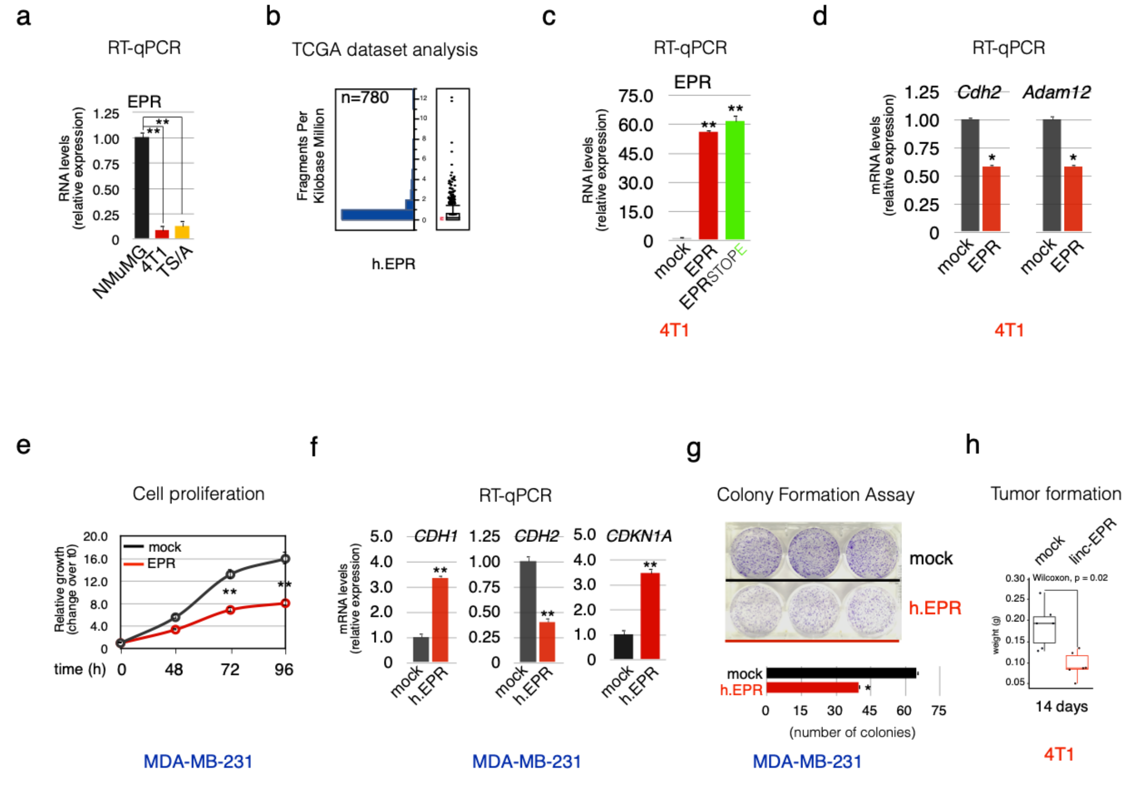

Rossi et al. Supplementary Figure 7

Supplementary Figure 7. EPR expression in transformed mammary gland cells. (Related to Figure 6).

(a) The expression of EPR was quantified by qRT-PCR analysis in the indicated cell types. (b) Distribution of h.EPR in the TCGA Breast Cancer (BRCA) dataset. (c) qRT-PCR analysis of EPR levels in mock, EPR or EPRSTOPE-overexpressing 4T1 cells as well as of h.EPR in either mock or EPR-overexpressing MDA-MB-231 cells. (d) qRT-PCR analysis of the indicated transcripts in either mock-transfected (mock) or EPR-overexpressing (EPR) 4T1 cells. (e) Cell proliferation analysis (using Crystal violet staining) of either mock or EPR (EPR) -overexpressing MDA-MB-231 cells. (f) qRT-PCR analysis of the indicated transcripts in either mock-transfected (mock) or human EPR-overexpressing (h.EPR) MDA-MB-231 cells. (g) Either mock or h.EPR-overexpressing MDA-MB-231 cells were seeded at low density in 6-wells plates and colony-formation assays were performed after 6 days. A representative (of three independent) plate is shown. (h) Tumors (n = 6 tumors/group, see Figure 6h) were removed and weighted at the end of the experiment (two weeks). Box plot analysis of tumor weight is shown. Box plots display summaries of the data distribution: the lower whisker is the minimum, the lower box edge is first quartile, the middle line is the median, the upper box edge is the third quartile, and the upper whisker the maximum value. Data were analyzed in

R version 3.4.3, using the statistic method Wilcoxon Unpaired Test as implemented in `stat_compare_means` in ‘ggplot 2.2.1’.

The values of qRT-PCR experiments shown are averages (±SEM) of three independent experiments performed in triplicate. The values of cell proliferation experiments (panel e) are averages (±SEM) of three independent experiments performed in triplicate. Statistical significance: *p< 0.01, **p < 0.001 (Student’s t-test).

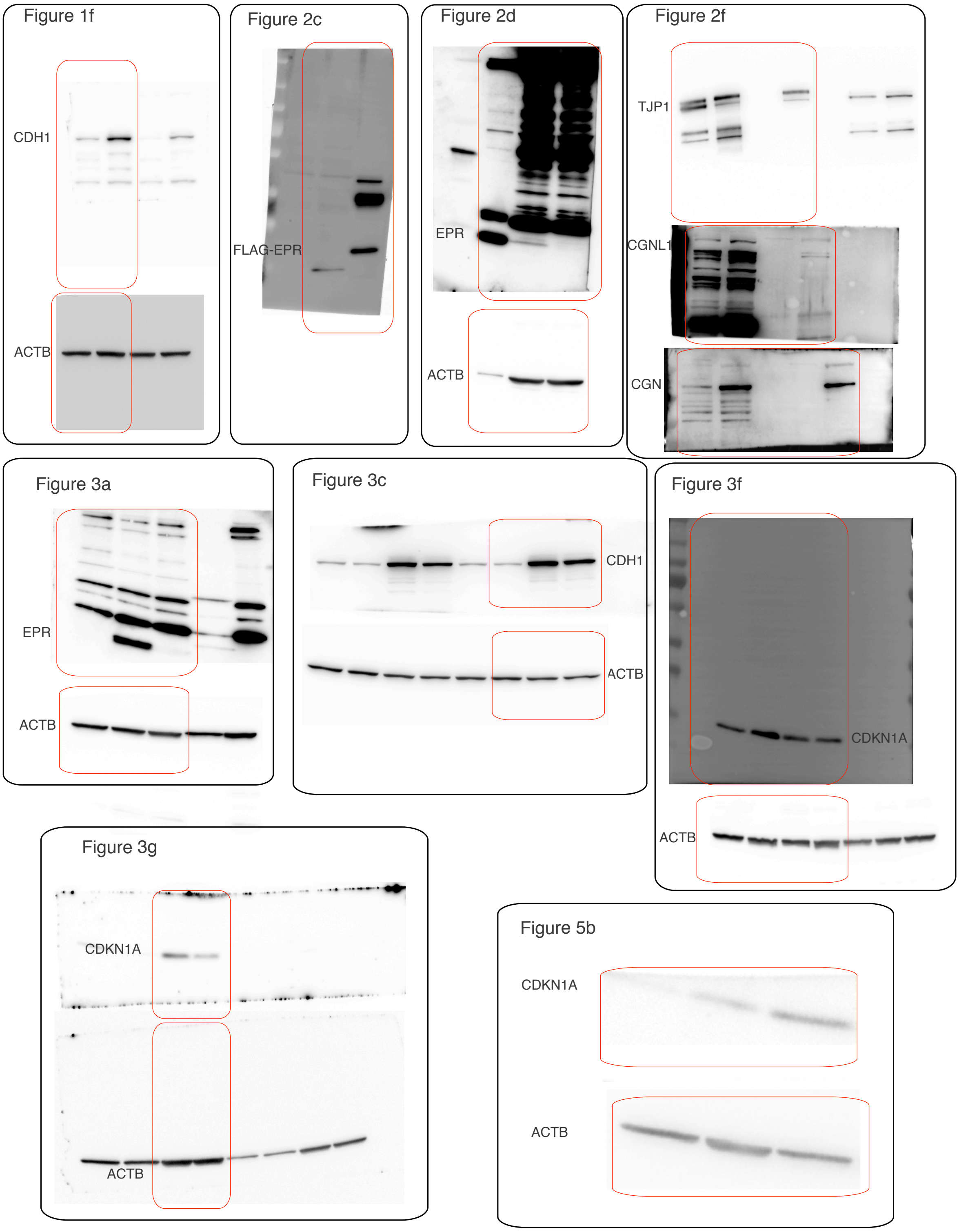

<u>Supplementary Figure 8. Uncropped and unprocessed scans of the Immunoblots presented in the Figures of this study</u>.

The lanes of each Immunoblot that are displayed in the paper are indicated by red boxes.

Supplementary Table 1a.

RNA-Seq experiments indicated that TGF-β treatment (24 hours) modulates (llog2 fold changesl >2.0, p<0.01) the expression of 304 lncRNAs in NMuMG cells. EPR (BC030870 is the official gene name) is highlighted in yellow.

Official Gene Name	Coef. TGFb-treated vs. Control-treated NMuMG cells	p value TGFb-treated vs. Control-treated NMuMG cells
Gm10451	7.092	0
Gm16564	6.433	0
Gm14002	5.43	0
1700025N21Rik	5.325	0
Gm13861	5.323	0
Gm5424	5.317	0
Gm2022	5.215	9.00E-05
Gm2016	5.179	0
Gm13986	5.179	1.00E-05
8030451A03Rik	5.145	0
Gm8332	4.992	0
Gm12153	4.929	0
5330416C01Rik	4.883	0
5031434C07Rik	4.759	0
Gm14492	4.737	0
Gm15958	4.58	0
Gm13889	4.541	2.00E-05
G630018N14Rik	4.504	2.00E-04
Gm11216	4.233	0
Gm11375	4.209	1.00E-05
D030025P21Rik	4.194	1.00E-04
9430029A11Rik	4.124	6.00E-05
4833422C13Rik	4.064	0
E230016K23Rik	4.054	3.00E-05
6030419C18Rik	4.006	0.00013
1700001F09Rik	3.997	6.00E-04

Supplementary Table 1b

RIP-Seq analysis revealed that the interaction of KHSRP with 67 lncRNA is modulated (llog2 fold changesl >2.0, p<0.01) by TGF-β treatment (**6 hours**).

EPR (BC030870 is the official gene name) is highlighted in yellow.

ENSEMBL gene I.D.	chromosome	start_position	end_position	strand	official gene name	TGFb-Control	FCrank	TGFb.RIP.C	TGFb.RIP.TGFb
ENSMUSG00000097125	17	29461896	29491925.00	-1	Gm26885	3.71	15687	-0.40	3.30
ENSMUSG97126	8	110754992	110758970.00	1	Gm15894	3.25	15653	-0.81	2.44
ENSMUSG97127	4	149904006	149908149.00	1	Gm13070	3.16	15648	-0.81	2.35
ENSMUSG97128	17	15375000	15379475.00	1	A930024N18Rik	3.04	15636	0.78	3.82
ENSMUSG97129	2	118123451	118124047.00	-1	Gm29233	2.91	15612	2.70	5.61
ENSMUSG97130	2	128195993	128429349.00	-1	Gm14005	2.90	15611	1.13	4.03
ENSMUSG97131	16	92612824	92620032.00	1	Gm26626	2.88	15609	-0.27	2.61
ENSMUSG97132	8	122269569	122272650.00	1	Gm22	2.85	15606	0.12	2.98
ENSMUSG97133	X	109001485	109002296.00	1	5730416F02Rik	2.82	15601	-0.27	2.55
ENSMUSG97134	18	66291274	66319607.00	1	Gm15958	2.65	15580	-0.40	2.24
ENSMUSG97135	16	35966752	35983230.00	-1	Gm15564	2.54	15563	1.34	3.87
ENSMUSG97136	4	130824394	130826313.00	-1	Gm26716	2.48	15559	1.01	3.49
ENSMUSG97137	7	89510007	89511368.00	1	Prss23os	2.37	15540	3.29	5.66
ENSMUSG97138	18	75365332	75367088.00	-1	Gm20544	2.36	15536	-0.03	2.33
ENSMUSG97139	9	3000282	3038313.00	-1	Gm26870	2.35	15532	4.25	6.60
ENSMUSG97140	6	67036599	67080654.00	1	E230016M11Rik	2.28	15520	1.75	4.04
ENSMUSG97141	7	25152457	25156627.00	1	D930028M14Rik	2.24	15514	-0.03	2.21
ENSMUSG97142	9	56223715	56223771.00	-1	Gm24270	2.13	15478	1.79	3.91
ENSMUSG97143	4	119133749	119138801.00	-1	Gm12868	2.05	15449	0.02	2.07
ENSMUSG97144	1	176835724	176836062.00	1	Gm38158	2.10	15436	2.66	4.66
ENSMUSG97145	2	129205958	129230579.00	-1	9830144P21Rik	2.00	15434	1.72	3.72
ENSMUSG97146	12	13455020	13455970.00	-1	Gm6736	2.00	15432	3.65	5.64
ENSMUSG97147	1	119529480	119536165.00	-1	3830432H09Rik	2.00	15429	0.35	2.34
ENSMUSG97148	15	85581142	85593708.00	1	AU022754	2.00	15426	1.46	3.44
ENSMUSG97149	16	11144125	11144181.00	1	Gm23935	2.00	15417	3.66	5.63
ENSMUSG97150	13	9834469	9834525.00	-1	Gm24187	2.00	15405	1.27	3.20
ENSMUSG97151	2	4834136	4834612.00	-1	Gm13176	2.00	15404	0.47	2.39
ENSMUSG97152	1	55444074	55445024.00	-1	Gm6644	2.00	15402	4.05	5.96
ENSMUSG97153	4	39895208	39896162.00	-1	Gm12387	2.00	15399	0.54	2.45
ENSMUSG97154	14	45587740	45598512.00	1	Gm15601	2.00	15383	0.98	2.82
ENSMUSG97155	10	24549441	24597108.00	-1	Gm15270	2.00	15377	7.12	8.95
ENSMUSG97156	11	40672906	40673856.00	-1	Gm12138	2.00	15360	2.92	4.70
ENSMUSG97157	4	63979854	64149924.00	1	8030451A03Rik	2.00	15329	1.92	3.62
ENSMUSG97158	8	25037052	25041757.00	1	Gm16933	2.00	15320	4.57	6.26
ENSMUSG97159	17	36111426	36117121.00	1	BC023719	2.00	15309	2.27	3.93
ENSMUSG97160	2	40127504	40127860.00	-1	Gm13453	2.00	15287	3.20	4.81
ENSMUSG97161	8	81197627	81198214.00	-1	Gm4899	2.00	15283	2.56	4.16
ENSMUSG97162	13	31626446	31626543.00	-1	Gm27516	2.00	15263	1.42	2.99
ENSMUSG00000102887	2	6132869	6140568.00	1	Gm10857	2.00	15230	0.90	2.40
ENSMUSG00000087341	6	108577039	108660934.00	-1	0610040F04Rik	2.00	15228	4.48	5.98
ENSMUSG00000095539	10	100144254	100144363.00	1	Gm25287	-2.00	727	6.53	4.96
ENSMUSG00000074398	3	96555765	96566801.00	-1	Gm15441	-2.00	700	5.71	4.11
ENSMUSG00000097493	15	60822271	60831793.00	1	9930014A18Rik	-2.00	592	7.80	6.05
ENSMUSG00000085715	X	103431517	103484957.00	1	Tsix	-2.00	559	4.04	2.24
ENSMUSG00000092341	19	5795690	5802672.00	-1	Malat1	-2.00	515	11.98	10.11
ENSMUSG00000085587	X	10717473	10718710.00	-1	Gm14493	-2.00	490	6.15	4.24
ENSMUSG00000097365	3	9403064	9437233.00	1	C030034L19Rik	-2.00	469	7.75	5.78
ENSMUSG00000091479	15	66929607	66931107.00	1	Gm17035	-2.00	459	7.25	5.26
ENSMUSG00000096972	2	169633646	169888503.00	-1	Gm26883	-2.06	426	5.82	3.76
ENSMUSG00000097060	8	126566970	126590988.00	1	Gm26759	-2.09	415	6.26	4.17
ENSMUSG00000082100	11	19720355	19721472.00	1	Glns-ps1	-2.34	311	6.88	4.54
ENSMUSG00000085083	11	101264762	101266303.00	-1	Gm11615	-2.44	285	0.30	-2.14
ENSMUSG00000097017	7	25816654	25820654.00	1	Gm26707	-2.56	256	0.26	-2.30
ENSMUSG00000103308	1	184610280	184629473.00	-1	Gm37800	-2.79	212	0.30	-2.48
ENSMUSG00000087484	5	144241690	144256186.00	-1	2900089D17Rik	-2.81	210	0.51	-2.30
ENSMUSG00000097124	3	121531619	121536206.00	1	A530020G20Rik	-3.02	174	0.71	-2.30
ENSMUSG00000097354	6	129208412	129237982.00	-1	2310001H17Rik	-3.20	136	0.71	-2.48
ENSMUSG00000091390	17	57041775	57047143.00	1	Gm17168	-3.31	117	1.01	-2.30
ENSMUSG00000085563	1	180952064	180958149.00	-1	2210411M09Rik	-3.52	94	0.30	-3.22
ENSMUSG00000086140	5	114968823	114971400.00	1	Hnf1aos2	-3.70	84	0.12	-3.58
ENSMUSG00000085918	4	140810993	140817511.00	1	Gm13032	-3.80	76	1.66	-2.14
ENSMUSG00000086265	13	4248735	4249025.00	1	Marcksl1-ps4	-3.98	64	1.29	-2.69
ENSMUSG00000081886	X	100585009	100585408.00	1	Gm6175	-3.98	63	6.94	2.95
ENSMUSG00000074300	8	65085613	65129563.00	-1	BC030870	-4.49	45	0.43	-4.07
ENSMUSG00000097805	15	42676260	42704616.00	1	Gm17473	-5.27	29	1.20	-4.07
ENSMUSG00000093662	9	74848463	74891476.00	1	Gm20649	-6.40	15	2.82	-3.58
ENSMUSG00000084074	X	66763382	66764574.00	1	Gm8310	-6.56	14	0.17	-6.39
