# Supplementary material for: LncRNA EPR controls epithelial proliferation by coordinating Cdkn1a transcription and mRNA decay response to TGF-β
Source: Nat Commun. 2019 Apr 29;10:1969. doi: 10.1038/s41467-019-09754-1 (PMC6488594; doi:10.1038/s41467-019-09754-1)
Supplement: Supplementary file 3 — Description of Additional Supplementary Files [file 41467_2019_9754_MOESM3_ESM.pdf]

Description of Additional Supplementary Files

File Name: Supplementary Data 1
Description: List of EPRp interactors. List of proteins that specifically interact with EPRp identified by mass spectrometry after immunoaffinity purification and SDS-PAGE analysis in NMuMG cells stably overexpressing FLAG-tagged EPRp.

File Name: Supplementary Data 2
Description: RNA-Seq analysis comparing the transcriptome of NMuMG mock cells with that of NMuMG cells overexpressing either EPR or EPRSTOPE. Experiments were performed in triplicate and analyzed as detailed in the Methods Section.

File Name: Supplementary Data 3
Description: List of primers used for qRT-PCR and qPCR analyses as well as of 3' Bio-TEG oligonucleotides used for ChIRP experiements.
