# Supplementary material for: LncRNA EPR controls epithelial proliferation by coordinating Cdkn1a transcription and mRNA decay response to TGF-β
Source: Nat Commun. 2019 Apr 29;10:1969. doi: 10.1038/s41467-019-09754-1 (PMC6488594; doi:10.1038/s41467-019-09754-1)
Supplement: Supplementary file 7 — Reporting Summary [file 41467_2019_9754_MOESM7_ESM.pdf]

nature research

Corresponding author(s): Alberto Inga, Paola Briata, Roberto Gherzi
Last updated by author(s): Mar 11, 2019

Reporting Summary

Nature Research wishes to improve the reproducibility of the work that we publish. This form provides structure for consistency and transparency in reporting. For further information on Nature Research policies, see Authors & Referees and the Editorial Policy Checklist.

Statistics

For all statistical analyses, confirm that the following items are present in the figure legend, table legend, main text, or Methods section.

n/a	Confirmed	
☐	☒	The exact sample size (*n*) for each experimental group/condition, given as a discrete number and unit of measurement
☐	☒	A statement on whether measurements were taken from distinct samples or whether the same sample was measured repeatedly
☐	☒	The statistical test(s) used AND whether they are one- or two-sided<br>*Only common tests should be described solely by name; describe more complex techniques in the Methods section.*
☒	☐	A description of all covariates tested
☒	☐	A description of any assumptions or corrections, such as tests of normality and adjustment for multiple comparisons
☐	☒	A full description of the statistical parameters including central tendency (e.g. means) or other basic estimates (e.g. regression coefficient) AND variation (e.g. standard deviation) or associated estimates of uncertainty (e.g. confidence intervals)
☐	☒	For null hypothesis testing, the test statistic (e.g. *F*, *t*, *r*) with confidence intervals, effect sizes, degrees of freedom and *P* value noted<br>*Give P values as exact values whenever suitable.*
☐	☒	For Bayesian analysis, information on the choice of priors and Markov chain Monte Carlo settings
☒	☐	For hierarchical and complex designs, identification of the appropriate level for tests and full reporting of outcomes
☐	☒	Estimates of effect sizes (e.g. Cohen's *d*, Pearson's *r*), indicating how they were calculated

Our web collection on statistics for biologists contains articles on many of the points above.

Software and code

Policy information about availability of computer code

Data collection	For RNA-Seq data: FASTX [http://hannonlab.cshl.edu/fastx_toolkit],
Data analysis	For transcript abundance analysis, the Kallisto 0.43.1 softwarewas used. In order to transform in Count per Million gene expression data we used the edgeR_3.16.5 and limma_3.30.13 tool. For Box plot analysis the ggpubr (https://CRAN.R-project.org/package=ggpubr) and ggplot2 (https://ggplot2.tidyverse.org) tools were used. Multiple alignment of mammalian EPR sequences was conducted by using the ClustalW2 package (http://www.clustal.org/clustal2/). For GO analysis, the web application EnrichR (http://amp.pharm.mssm.edu/Enrichr/) was used.

For manuscripts utilizing custom algorithms or software that are central to the research but not yet described in published literature, software must be made available to editors/reviewers. We strongly encourage code deposition in a community repository (e.g. GitHub). See the Nature Research guidelines for submitting code & software for further information.

Data

Policy information about availability of data

All manuscripts must include a data availability statement. This statement should provide the following information, where applicable:
- Accession codes, unique identifiers, or web links for publicly available datasets
- A list of figures that have associated raw data
- A description of any restrictions on data availability

Raw data from RNA deep-sequencing analyses have been published on the GEO archive under the Accession GSE113178 [https://www.ncbi.nlm.nih.gov/geo/query/acc.cgi?acc=GSE113178]. Human EPR expression in different subpopulations of FACS-sorted normal breast cells21 and in different human organs was inferred through either the Expression Atlas (https://www.ebi.ac.uk/gxa/home) or the GEPIA web server (http://gepia.cancer-pku.cn). Proteins interacting with EPRp were unambiguously identified by searching a comprehensive non-redundant protein database of the National Center for Biotechnology Information (NCBI, http://www.ncbi.nlm.nih.gov/) and the Mass Spectrometry protein sequence DataBase (MSDB, http://msdn.microsoft.com/en-us/library/ms187112.aspx).

Field-specific reporting

Please select the one below that is the best fit for your research. If you are not sure, read the appropriate sections before making your selection.

☒ Life sciences ☐ Behavioural & social sciences ☐ Ecological, evolutionary & environmental sciences

For a reference copy of the document with all sections, see nature.com/documents/nr-reporting-summary-flat.pdf

Life sciences study design

All studies must disclose on these points even when the disclosure is negative.

Sample size	The number of BALB/c mice for each experimental group was calculated taking into account: an hypothesized 40% reduction of the tumor mass size (based on our previous in vitro studies), statistical significance of $p<0.05$ (Wilcoxon test), statistical power: 0.8, estimated deviation: 20%.
Data exclusions	No data were excluded
Replication	All replications were successful
Randomization	For experiments involving mice the allocation of animals to different experimental treatments was randomized.
Blinding	For RNA-Seq experiments the investigators that performed data analysis were blinded to cell type allocation. For experiments involving mice, the investigators were blinded to group allocation during data analysis.

Reporting for specific materials, systems and methods

We require information from authors about some types of materials, experimental systems and methods used in many studies. Here, indicate whether each material, system or method listed is relevant to your study. If you are not sure if a list item applies to your research, read the appropriate section before selecting a response.

Materials & experimental systems

n/a	Involved in the study	
☐	☒	Antibodies
☐	☒	Eukaryotic cell lines
☒	☐	Palaeontology
☐	☒	Animals and other organisms
☒	☐	Human research participants
☒	☐	Clinical data

Methods

n/a	Involved in the study	
☒	☐	ChIP-seq
☒	☐	Flow cytometry
☒	☐	MRI-based neuroimaging

Antibodies

Antibodies used	Anti-CDH1 goat polyclonal antibody (sc-31020, used at 1:500 final dilution), anti-CDKN1A mouse monoclonal antibody (sc-6246, used at 1:200 final dilution), and anti-HDAC1 rabbit polyclonal antibody (sc-7872, used at 1:500 final dilution) were from Santa Cruz; anti-TJP1 rabbit polyclonal antibody (ab96587, used at 1:100 final dilution), anti-SMAD3 rabbit polyclonal antibody (ChIP grade ab28379), anti-GFP rabbit polyclonal antibody (ChIP grade ab90, used at 1:200 final dilution) were from Abcam; mouse monoclonal anti-FLAG (F1804, used at 1:500 final dilution), mouse monoclonal anti-TUBA (DM1, used at 1:1000 final dilution) and mouse monoclonal anti-ACTB (AC-74, used at 1:30000 final dilution) were from Sigma Aldrich. Mouse monoclonal anti-RNA Polymerase II (clone CTD4H8) and rabbit polyclonal antibody to H3K27me3 (CS200603) were from Millipore. Rabbit polyclonal anti-CGN serum (C532, used at 1:5000 final dilution) against a purified recombinant 50 kDa C-terminal fragment of chicken cingulin as well as anti-CGNL1 rabbit polyclonal antibody (20893, used at 1:100 final dilution) were raised at the University of Geneve. Anti-EPR polyclonal rabbit antibody was generated by injecting rabbits with recombinant purified EPR expressed in E. Coli using the pQE-EPR at Cambridge Research Biochemicals (Billingham, Cleveland, UK).
Validation	For commercial antibodies, please see the websites of the manufacturers; for anti-CGN please see Citi S, Sabanay H, Kendrick-Jones J, Geiger B. Cingulin: characterization and localization. J Cell Sci. 1989 May;93 (Pt 1):107-22; for anti-CGNL1 please see Paschoud S, Yu D, Pulimeno P, Jond L, Turner JR, Citi S. Cingulin and paracingulin show similar dynamic behaviour, but are recruited independently to junctions. Mol Membr Biol. 2011 Feb;28(2):123-35. Anti-EPRp polyclonal rabbit antibody was generated by injecting rabbits with recombinant purified EPR expressed in E. Coli using the pQE-EPR at Cambridge Research Biochemicals (Billingham, Cleveland, UK). Experimental controls for anti-EPRp are provided in this studt.

Eukaryotic cell lines

Policy information about cell lines

Cell line source(s)	Murine immortalized NMuMG cells (ATCC, no. CRL-1636), 4T1 mouse mammary gland cancer cells (obtained from ATCC, no. CRL-2539), human mammary gland adenocarcinoma cells MDA-MB-231 (obtained from DSMZ, Germany, through Dr. G. Fronza, authenticated by STR DNA profiling), and human HEK-293 cells (ATCC, no. CRL-1573).
Authentication	MDA-MB-231 were authenticated by STR DNA profiling.
Mycoplasma contamination	All cell lines were tested for Mycoplasma contamination and resulted negative.
Commonly misidentified lines (See ICLAC register)	HEK-293 cells were used to verify transient expression of FLAG-tagged EPRp based on their highly efficient transfectability

Animals and other organisms

Policy information about studies involving animals; ARRIVE guidelines recommended for reporting animal research

Laboratory animals	BALB/c mice, 8–10-week-old, female (from Envigo)
Wild animals	The study did not involve wild animals.
Field-collected samples	The study did not involve field-collected samples.
Ethics oversight	All procedures involving animals have been approved by Institutional Animal Welfare Body (O.P.B.A.) and complied with the national current ethical regulations regarding the protection of animals used for scientific purpose (D. Lvo March 4th, 2014, n. 26, legislative transposition of Directive 2010/63/EU of the European Parliament and of the Council of September 22, 2010 on the protection of animals used for scientific purposes).

Note that full information on the approval of the study protocol must also be provided in the manuscript.
